# Supplementary material for: 3D Printed Bioreactor Enabling the Pulsatile Culture of Native and Angioplastied Large Arteries
Source: Front Cardiovasc Med. 2022 Jun 21;9:864580. doi: 10.3389/fcvm.2022.864580 (PMC9253513; doi:10.3389/fcvm.2022.864580)
Supplement: Supplementary file 4 [file Data_Sheet_1.docx]

**Supplementary Figures and Figure Legends**

**
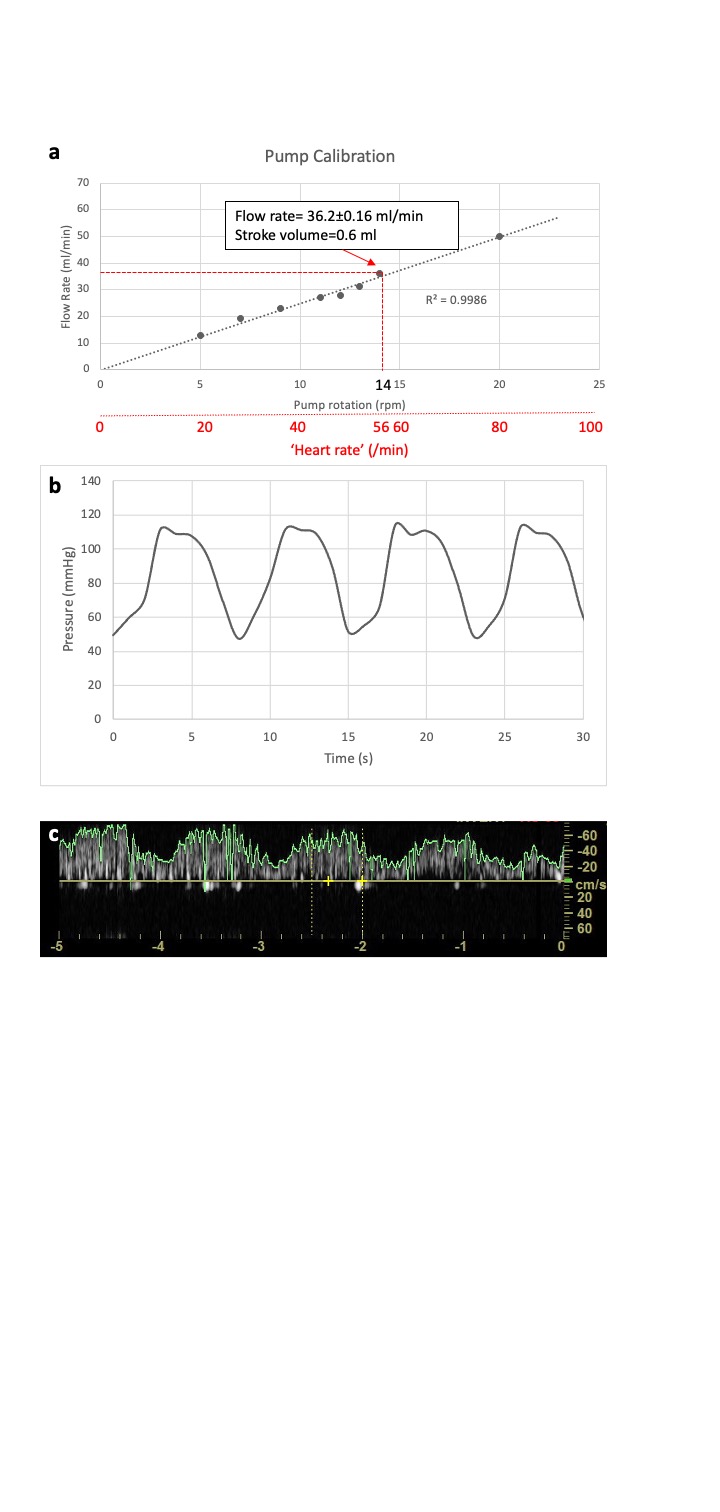
**

**Supplementary Figure 1. Perfusion Parameters**

Pump calibration was performed by measuring the weight of dispensed water over time, in correspondence to different pump speeds, and relative ‘heart rate’. These data were used to calculate the volumetric flow rate and stroke volume in relation to the different pump settings (a). To monitor the pressure changes in the system, a sensor was placed downstream of the artery. Pressure readings were acquired every second and plotted to observe pressure fluctuation (b). Doppler ultrasound measurement of the flow velocity in the artery shows systolic and diastolic variation (c).

**Supplementary File Legends**

**Supplementary File 1. Design file for manufacturing.** Computer assisted design file generated by Autodesk Fusion 360. File contain 3D models of the EasyFlow insert suitable for manufacturing. Dimensions of the model are defined in millimeter (mm) and are saved in the true size of the model. Perfusion insert and adaptor were merged in a single object to be manufactured as a singular unit.

**Supplementary Video 1. 3D rendered animated model of perfusion system.** Complete and assembled EasyFlow system has been recreated in a 3D environment with Autodesk Fusion 360. The resulting model was used to generated an animated video breaking the system down to its core components.

**Supplementary Video 2. Ultrasound recording of perfused tissue.** Doppler ultrasound recording of perfused carotid artery tissue following 7 day culture in EasyFlow. Online measurements were acquired non-invasively. Recordings show the local flow velocity (cm/s), lumen dimensions (mm) and a B-mode recording, highlighting the vessel wall movement and composition.

**Supplementary Video 3. Ultrasound recording of injured tissue.** Doppler ultrasound recording of balloon carotid artery tissue following 7 day culture in EasyFlow. Online measurements were acquired non-invasively. Recordings show the local flow velocity (cm/s), lumen dimensions (mm) and a B-mode recording, highlighting the vessel wall movement and composition.
